# What breaks the flow of reading? A study on characteristics of attentional disruption during digital reading

**DOI:** 10.3389/fpsyg.2022.987964

**Published:** 2022-10-12

**Authors:** Guillaume Chevet, Thierry Baccino, Annie Vinter, Véronique Drai-Zerbib

**Affiliations:** ^1^Laboratory LEAD-CNRS, UMR5022, Université Bourgogne, Dijon, France; ^2^LUTIN, University Paris 8, Paris, France

**Keywords:** reading, attentional disruption, interruption, mind wandering, multitasking

## Abstract

Reading is increasingly taking place on digital media, which are vectors of attentional disruption. This manuscript aims to characterize attentional disruption during reading on a computer screen in an ecological environment. To this end, we collected information relating to reader interruptions (number, type, duration, position, mental effort, and valence) and *self-caught mind wandering* (occurrence, position) throughout the reading session for *high* and *low* media multitaskers in their own specific ecological environment, at home. Comprehension of the narrative text was assessed both with surface and inferential questions. In total, 74 participants (*M* = 22.16, SD = 2.35) took part in the experiment. They reported attentional disruptions on average every 4 mins during reading. Moreover, there were more attentional disruptions during the first half of the text. Most interruptions were short and little mental effort was required to process them. We made a distinction between media-related and media-unrelated related interruptions. Multiple linear regression analyses showed that media-unrelated interruptions were actually related to better performance for both inferential and surface level questions. Furthermore, media-related interruptions were more frequent for *high* than *low* media multitaskers. Pleasure experienced when reading the text was also a significant predictor of comprehension. The results are discussed with regard to *Long-Term Working Memory* and strategies that the readers could have implemented to recover the thread of their reading.

## Introduction

Reading is not only a leisure activity but also one of the main vectors of knowledge transmission in education. Today, at a time when reading is increasingly done on digital media such as computers or tablets, cognitive psychology is taking an interest in the impact of these digital reading media on text comprehension. Many studies have reported that digital media reduce reading comprehension compared to paper (Delgado et al., 2018; Kong et al., 2018; Delgado and Salmerón, 2021). The characteristics of the medium and the change in reading practices they bring about are the causes of this decrease in performance (Baccino and Drai-Zerbib, 2015). Since multiple tasks can be managed on the same digital medium, reading can be interrupted by the reader or by an external prompt (Fox et al., 2008; Bowman et al., 2010; Subrahmanyam et al., 2013; Tran et al., 2013). This may be the case when they read on a computer connected to the Internet or a smartphone for example; in this situation pop-ups (commercial or related to a notification) may occur and disrupt reading. The reader can also interrupt his reading activity to engage another activity proposed by these media. These interruptions could affect reading at different levels: comprehension, reading experience or reading satisfaction. In addition, the ability to perform multiple activities on the same medium as that used for reading could also promote situations in which the reader’s attention may shift away from the text, even as the eyes continue to move over the lines. This phenomenon of mindless reading, first mentioned by Rayner and Fischer (1996), is also referred to as *zoning out* or *mind wandering* during reading (Schooler et al., 2004; Smallwood, 2011).

Although various fields of psychology have investigated attentional disruptions in reading in the laboratory setting, little is known about their occurrence, nature, and effects on reading in more ecological settings. The primary goal of this study was to quantify and identify situations in which attention was diverted away from the text during a computer reading session in the context of the reader’s natural environment. Unlike laboratory settings in which the sources of distraction are limited as much as possible or introduced artificially, collecting data from an ecological environment provides information on attentional disruptions that occur in real situation. The exploratory approach of our study combined a classical experimental design and a survey method to analyze the effects of several forms of attentional disruption on reading comprehension and satisfaction.

### Reading comprehension

According to the well-known construction/integration model (Van Dijk and Kintsch, 1983; Kintsch, 1991), text comprehension requires the incremental building of a text representation at three levels: surface, semantic and referential. At the surface level, readers access lexical and syntactic information. At the semantic level, they attempt to associate the information contained in the various propositions in the text to achieve local coherence. At the referential level, a link has to be established between information from different parts of the text, and the reader’s personal knowledge. A global coherence, calculated from the situation model which represents the information evoked by the text and stored in memory, must be elaborated from this referential level. However, this reading comprehension approach is essentially based on a propositional analysis of the text and does not describe the role of perception or attentional processes in the building of reading comprehension. However, as the cognitive system has only limited attention and working memory resources (Miller, 1956; Kahneman, 1973; Cowan et al., 2004; Wickens, 2008), the construction/integration model acknowledges that reading involves selecting and integrating the most relevant elements in order to build the situation model (Kintsch, 1998). Thus, reading involves both selective attention (Treisman, 1964; Broadbent, 2013) and sustained attention (Posner and Boies, 1971; Posner and Petersen, 1990) in order to continuously elaborate text meaning through the three levels described above (Schooler et al., 2004; Smallwood et al., 2008; Smallwood, 2011; Arrington et al., 2014).

### Attentional disruption in reading

**FIGURE 1:**
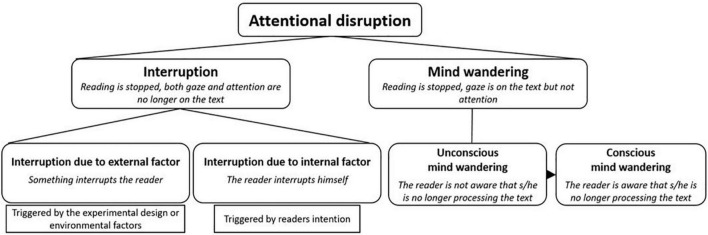
Attentional disruptions in reading can be divided into two categories. The first category is interruptions: in this case, both gaze and attention are no longer focused on the text. Interruptions can be due to external or internal factors. Interruptions due to external factors are triggered by the experimental design or environmental factors. Interruptions due to internal factors are intentionally brought about by readers. The second category is mind wandering: in this case, the gaze is still on the text but without focused attention. Readers may not initially be aware that they are mind wandering but they may also eventually notice it.

There are two possible configurations in which attention is no longer focused on the text (Figure 1). The first occurs when reading is interrupted (Glanzer et al., 1981, 1984; McNamara and Kintsch, 1996; Oulasvirta and Saariluoma, 2006; Cho et al., 2015; Foroughi et al., 2015). When reading is interrupted, either intentionally or due to an external factor, attention switches to the distracting task. The second occurs when readers experience a *mind wandering* episode (Schooler et al., 2004; Reichle et al., 2010; Smallwood et al., 2008; Smallwood, 2011; Feng et al., 2013), in which they are distracted by thoughts that are unrelated to the text (Murray et al., 2020). In this scenario, their eyes continue to move over the text (Reichle et al., 2010), while their attention is focused on thoughts unrelated to it.

#### Interruptions due to external factors

The effect of interruptions in reading has been extensively studied in the laboratory, often using tasks that are not particularly ecological to induce interruptions. Using interfering reading or arithmetic tasks, Glanzer et al. (1981, 1984) and Glanzer and Nolan (1986) observed a slower sentence reading speed following the interruption, without any impairment to reading comprehension. However, the decrease of reading speed was no longer observed when readers were able to reread the sentence that preceded the interruption (Glanzer et al., 1984), were reminded of the topic of the text (Lorch, 1993), or could see an image representing a scene described in the text (Schneider and Dixon, 2009). Ericsson and Kintsch (1995) interpreted this lack of effect of the interruption on reading comprehension as support for their Long-Term Working Memory theory. This theory considers that the information read is instantly integrated and preserved in long-term working memory (Ericsson and Kintsch, 1995; Delaney and Ericsson, 2016), even if short-term working memory is not available. In this view, the increased reading time devoted to the post-interruption sentence permits the reactivation of the situation model, thus resulting in preserved performance. The results of other studies support this theory by showing no impairment in comprehension, irrespective of whether the interruption occurred within a sentence (McNamara and Kintsch, 1996; Chevet et al., 2021), due to a frequent and difficult interruption task (Oulasvirta and Saariluoma, 2006, or because of an interfering arithmetic or reading task (Cho et al., 2015). However, in two recent studies, Foroughi et al. (2015, 2016a,2016b) and Delaney and Ericsson (2016) found an impairment of text comprehension when the questions testing comprehension required the participants to connect and synthetize information across the text in order to provide the right answer. They emphasized the need to assess a deep instead of a surface comprehension of the text.

Some laboratory studies have used interruptions more similar to those occurring in a real situation, such as, for example, the use of instant messaging (Fox et al., 2008; Bowman et al., 2010; Tran et al., 2013) or multitasking during a reading task (Subrahmanyam et al., 2013). Most of these studies have not shown any effect on reading comprehension. To the best of our knowledge, only one study (Kononova et al., 2016), found a decrease in recognition of information from the text when participants were forced to switch between reading an online article and checking their Facebook page. However, in this study, the presentation of the text was experiment-paced. This situation did not allow participants to reread the text after the interruption, especially when the pace was too fast for them. Such an experimental design could explain the decrease in performance (Oulasvirta and Saariluoma, 2006; Bowman et al., 2010; Cane et al., 2012; Chevet et al., 2021; Clinton-Lisell, 2021).

#### Interruptions due to internal factors

Moreover, it is worth noting that because the interruptions in laboratory settings are mainly triggered by the experimental design, it is impossible to identify intentional sources of interruption during reading, for example when one decides to take a coffee break in the middle of reading an article. However, such interruptions sometimes lead to a greater impact on the main task than external interruptions (Mark et al., 2005), hence the importance of taking them into consideration. Some studies have tried to identify the source of distraction while participants performed a learning task in an ecological environment. They arranged the experimental room to look like a student’s learning environment (Subrahmanyam et al., 2013; Calderwood et al., 2014; Deng, 2020) and instructed participants to act as they would do at home in order to identify *task-switching* behaviors. These methods are very effective for gathering information about what participants are doing in this organized space, including intentional interruptions. However, they can only represent a rough sketch of what they actually do in their own environments. Another approach to collecting data that reflects students’ natural behaviors with their computer in class has been proposed by Kraushaar and Novak (2010). In their study, the behavior of students was collected using spyware (monitoring software) installed on their laptops to gather information about what they were doing during a lecture. This method makes it possible to collect very precise information concerning the use of the computer but does not allow researchers to gather information regarding the use of other media or information relating to other sources of distraction.

#### Mind wandering while reading

The second situation in which attention is disconnected from reading is known as *mind wandering* (Schooler et al., 2004; Smallwood, 2011; Feng et al., 2013). In this case, the readers are thinking of something unrelated to the text while their eyes continue to scan the page. They may or may not be aware of this discrepancy between what their eyes are looking at and what their cognitive system is processing (Schooler et al., 2004). There is some indication that this discrepancy could lead to impaired reading comprehension (Unsworth and McMillan, 2013). *Mind wandering* could affect the reading process. Indeed after *mind wandering* readers engage in re-reading behavior almost half of the time (Varao-Sousa et al., 2017). Two experimental paradigms are usually used to assess *mind wandering* in laboratory settings (Smallwood and Schooler, 2006; Smallwood et al., 2008; Varao-Sousa and Kingstone, 2019). *Self-caught mind wandering* needs the readers to become aware of the disconnection between the content of their thought and the content of the text and to report this (Varao-Sousa and Kingstone, 2019). *Probe-caught mind wandering* involves interrupting them and asking them to report if they were processing the text or not. This second method is efficient for catching *mind wandering* and provides a better estimation of overall *mind wandering* frequency (Smallwood and Schooler, 2006). However, it makes it necessary to interrupt the readers. It is important to emphasize that *mind wandering* is not itself an internal interruption but precedes it. Previous study comparing reading on paper vs. screen shows that mind wandering is more present when reading on screen than paper under time pressure but not under free time (Delgado and Salmerón, 2021). However, little is known about the occurrence of mind wandering while reading on screen in an ecological environment at home. In such situation, the ability to perform multiple activities on the computer could promote situations of mind wandering.

### Our study

The aim of the present study is to identify and quantify the attentional disruptions that occur while reading a text online, when readers are in the context of their usual reading environment, at home; a context in which there are many sources of distractions (smartphone, television, family member, pet, etc.). This study also assesses the impact of disruptions on reading comprehension and reading experience.

We organized a long, supervised session in which participants read a narrative text at home. Participants were required to self-report the occurrence of interruptions and their characteristics (type, duration, position, mental effort, and valence) on a grid that we had developed ourselves. To our knowledge, no study has sought to identify and quantify the actual interruptions encountered by readers during a reading session in an ecological environment and their relation to reading comprehension and reading satisfaction. Indeed, in the previous studies mentioned above, the types of interruption and their occurrence were systematically included in the experimental design. Our approach allowed us to gather information on both internal and external sources of interruptions. In order to collect data regarding self-reported *mind wandering* during the task, we asked readers to *press* the central key “G” of the keyboard. This method is able to provide a correct estimation of *mind wandering* in an ecological situation (Varao-Sousa and Kingstone, 2019). As the core objective of our study was to quantify and identify actual disruptions, we wanted to avoid the use of the *probe-caught* methodology, which implies external interruptions.

Because the literature reports rather different results for inferential and surface questions (Cho et al., 2015; Foroughi et al., 2015), reading comprehension was assessed by means of multiple-choice questions including factual and inferential information. Moreover, we wanted to observe whether the individuals who were most likely to multitask when using media during their daily activities were also those who tended to be interrupted or interrupted themselves more regularly during their media use. We used a media multitasking index (Ophir et al., 2009) to assess the number of media simultaneously used by participants.

We expected a negative correlation between the number of interruptions and the scores on inferential questions, but not on surface question. Adopting an exploratory approach, we assessed these relationships for several types of attentional disruption (*mind wandering*, internal interruption, and external interruption). In addition, different aspects of reading experience were evaluated by means of questions which the participants responded to on Likert scales. We then evaluated the relationship between attentional disruptions and the pleasure experienced when reading the text, and between the pleasure experienced when reading the text and comprehension. A previous study shown that reading comprehension can be influenced by the motivation of reading (Guthrie and Cox, 2001). Thus, we should observe a positive correlation between pleasure when reading the text and reading comprehension. Moreover, a decrease in the pleasure experienced when reading the text as attentional disruption increased is expected.

Moreover, we compared *high* and *low media multitaskers* on the basis of the interruptions they experienced. In particular, we compared the number of interruptions related or not related to media. Media-related interruptions involved a medium such as a smartphone or a computer. Media-unrelated interruptions were those that did not involve any media. We expected that participants who reported being more likely to engage in multitasking with media during their activities would be those who were most frequently occupied by media-related interruptions during the experimental reading session. Therefore, *high-media multitaskers* should report more media-related interruptions than *low media multitaskers*. *High* and *low media multitaskers* were also compared on the basis of reading comprehension. We also tested the relation between the source of the interruption (media-related or media-unrelated) and reading comprehension.

## Materials and methods

### Participants

In total, 74 participants were recruited for this study (57 females, *M*_*age*_ = 22.16 years; *SD* = 2.35). All participants were native French-speakers and none had a language or attention disorder or suffered from dyslexia. Two participants were removed from the data because their error rate in reading comprehension was above 80%, suggesting that they were not engaged in the task. Three participants were removed because they did not complete the grid correctly. Our final sample therefore included 69 participants. Participants had all completed high school, on average they had completed 2.23 years of university studies. All participants freely gave their informed consent in accordance with the Declaration of Helsinki. A full review for ethical approval was not required according to our institution’s guidelines and national regulations.

### Apparatus

Participants used their own devices for the experiment in order to read the text and report interruptions. Even if the devices differed from participant to participant, the reading condition was always similar. The participants were instructed to read the text on a computer equipped with a microphone and an audio device. They were also required to activate their internet connection in order to (1) be directed to a web-based experiment built using Qualtrics, a software platform for experience management which requires the use of a web browser to collect survey data online, and (2) communicate *via* Teams with the experimenter if any question should arise during reading. The texts, the reading comprehension questions, and the grid used to report interruptions were all presented using Qualtrics. To record *self-caught mind wandering*, a JavaScript was developed and incorporated in the Qualtrics environment. This counted the number of times a participant pressed the “G” key on the keyboard for each page.

### Grid for reporting interruptions

**FIGURE 2:**
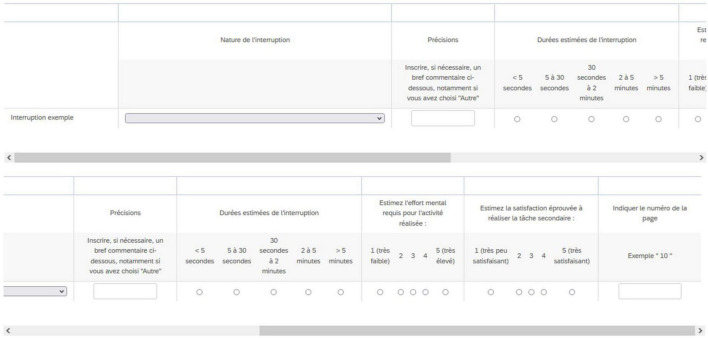
Screenshot of the grid in the Qualtrics environment. Due to its size, the grid was not displayed in full screen, participants had to scroll sideways (from ***left*** to ***right***) to see the different boxes to be filled. The first part is visible on the top image, and the rest on the bottom image.

To make it easier to report interruptions and their characteristics, we developed a grid in the Qualtrics environment (Figure 2). This grid was open in a different browser window from the text and allowed the participants to enter the information relating to the interruptions in six columns as detailed below:

– Type of interruption (pre-recorded choices).
– Free choice of interruption type “other” (reason for the interruption).
– Time spent on the interrupting task (Thresholds were chosen to facilitate the reporting of information by the participant: <5 s, 5–30 s, 30 s to 2 mins, 2–5 mins, >5 mins).
– Mental effort required during the interrupting task (on a 5-point Likert scale from 1 “*very low*” to 5 “*very high*”).
– Valence of the interrupting task (on a 5-point Likert scale from 1 “*very annoying*” to 5 “*very pleasant*”).
– The number of the page being read when the interruption occurred.

### Linguistic material

Two Sherlock Holmes novels were combined and used as reading material: *“The final problem”* and *“The Adventure of the Empty House,”* both by Arthur Conan Doyle. In the French version, the total length of these texts was 15,757 words, divided into 36 pages in our experimental setup. The participants confirmed that they had never read these novels. They were chosen because they require readers to produce many inferences in order to understand the story. In addition, pre-tests had shown that reading these novels took on average more than an hour, a time sufficiently long to make it probable that interruptions and *mind wandering* would occur.

Comprehension was evaluated by means of 50 multiple-choice questions. The participants had to select a response out of the four proposed, or “*I don’t know*”. In total, 22 questions required the participants to identify explicit information from the texts. We refer to these as surface questions. In total, 28 questions required them to connect information from different parts of the texts to produce new information. We refer to these as inferential questions. Reliability for total of questions was excellent (ω = 0.93), good for surface questions (ω = 0.83) and inferential questions (ω = 0.89).

### Pre-test

To evaluate the mean number of media used concurrently by the participants in their daily activities, we calculated a media multitasking index (MMI) adapted from Ophir et al. (2009). Our version of the questionnaire addressed 14 different media activities (reading print media, reading on computer, television, computer video, music, video games, phone calls, instant messaging on computer, instant messaging on phone, email, web surfing, social media, using computer software, and doing homework). The participants reported the mean number of hours they used each medium. They also filled out a matrix relating to media multitasking indicate how frequently they concurrently used other media (never, occasionally, regularly, and always) when engaged with a main medium. To calculate the MMI, the participants’ responses were converted into the numeric values: 0, 0.33, 0.66, and 1, respectively. The index was calculated using the formula provided by Ophir et al. (2009):


$$M\,M\,I=\sum_{i=1}^{14}\frac{M_{i}\times H_{i}}{H_{t\,o\,t\,a\,l}}$$

Where *M_*i*_* is the number of other media typically used when using the main medium, H*_*i*_* the number of hours per week using the main medium and *H*_*total*_ the total number hours per week using all the media. Based on the media multitasking index (MMI) score, the participants were divided into two groups, namely the “*high media multitaskers*” (score above the median) and the “*low media multitaskers*” (score below the median).

### Post-test

After reading and before responding to the reading comprehension questions, the participants had to report several items of information regarding their reading experience on a 5-point Likert scale going from 1 to 5. This information related to the pleasure they experienced when reading the text, the difficulty they experienced when reading the text, whether they felt that they had understood the story, and whether they felt they had been attentive. The reliability of this questionnaire was acceptable (α = 0.77). Participant had to indicate the number of reading comprehension questions they thought they had answered correctly. We calculated the difference between the actual score on comprehension question and this estimation as an indicator of self-evaluation.

### Procedure

The participants performed the experiment in their own environment. After a recruitment phase, the experimenters contacted the participants using the Microsoft Teams videoconferencing software. After scheduling the call, they sent them a personal Qualtrics link to allow them to access the experiment. The experimenter remained available remotely throughout the entire experiment in order to answer any questions from the participants and ensure that the experiment was carried out correctly.

After reading the instructions, the participants had to complete a questionnaire that collected socio-demographic information and their media utilization habits. They then had to read a text and answer the reading comprehension questions. They were instructed to read the text in their usual reading environment. They were recommended to read at their own pace, one page at a time, and to feel free to take as many breaks as necessary, as long as they read the text in only one time. There was no time limit for answering the post-test (Likert scale of 1–5) and reading comprehension questions. Finally, as high media multitaskers usually overestimate their performances (Ophir et al., 2009), participants were asked to estimate the number of reading comprehension questions they thought they had answered correctly.

At the same time as reading, the participants had to identify and report all the interruptions that occurred during the reading session in the dedicated grid. The link to the grid used to collect information about the interruptions was inserted in the instructions. When participants clicked on this link, they were directed to a tutorial that explained what to indicate in the grid, and how to complete it. They had to complete the tutorial before reading the texts in order to make sure they understood its use. They were also told to *“Press the G key on the keyboard each time you experience a period of inattention but without actually stopping reading. In other words, a period when you realize that you are no longer thinking about what your eyes are looking at (the text) but about something else (your shopping, your weekend night out, etc.)*” in order to collect episodes of self-reported *mind wandering*. By way of a reminder, the instructions to report interruptions and to press the “G” key were displayed at the top of each page. The reading time was recorded for each page. The participants were naive regarding the purpose of the experiment. However, we explained them that we were conducting a research on interruption during reading in the context of the reader’s natural environment and thanked them for their time dedicated to the study, at the end of the experiment.

### Classification of interruptions

The interruptions were categorized using the information reported in the grid. Following Deng (2020), a distinction was made, on the basis of the answers provided by the participants, between interruptions initiated by the participants themselves (internal) and interruptions caused by an external factor (external). We also distinguished between media-related and media-unrelated interruptions. Media-related interruptions were those that involved media use, while all other forms of interruptions were considered to be media-unrelated interruptions.

## Results

### Information relative to the reading session

Matched-sample *t-tests* used to compare the number of self-reported internal and external interruptions indicated in the grid showed that the difference was not significant, *t* < 1, *ns* (see Table 1). There was also no difference between the number of media-related interruptions and media-unrelated interruptions *t* < 1, *ns* (see Table 1). We used matched-sample *t-tests* to compare the number of interruptions and of *mind wandering* reported by participants between the first half of the text (pp. 1–18) and the second half of the text (pp. 19–36). Participants identified more interruptions in the first half of the text than in the second, *t*(68) = 7.60, *p* < 0.001, *d*′ = 0.91 (see Table 1). They also listed more episodes of *mind wandering* for the first part of the text than for the second part, *t*(68) = 6.57, *p* < 0.001, *d*′ = 0.79 (see Table 1). The distribution of the duration of the reading sessions was check with a Shapiro-Wilk test, the distribution was normally distributed with *W* = 0.97, *p* = 0.15.

**TABLE 1:** Descriptive data relative to the reading session.

	*M*	*SD*
Duration of the reading session (s)	5,020	1,159
Internal interruptions	3.52	2.48
External interruptions	3.62	3.13
Interruptions (pp. 1–18)	4.90	3.35
Interruptions (pp. 18–36)	2.26	1.84
Media-related interruptions	3.45	2.90
Media-unrelated interruptions	3.68	3.33
Total interruptions	7.19	4.56
Episodes of mind wandering reported (pp. 1–18)	9.48	7.88
Episodes of mind wandering reported (pp. 18–36)	5.12	5.28
Total episodes of mind wandering reported	14.60	12.70

#### Duration of the interruptions

A repeated-measures ANOVA with number of interruptions as the dependent variable and duration (<5 s, 5–30 s, 30 s to 2 mins, 2–5 mins, >5 mins) as within-subject factor showed a significant effect of reported duration, *F*(4, 272) = 39.80, *p* < 0.001, η^2;^*_*p*_* = 0.37.

**FIGURE 3:**
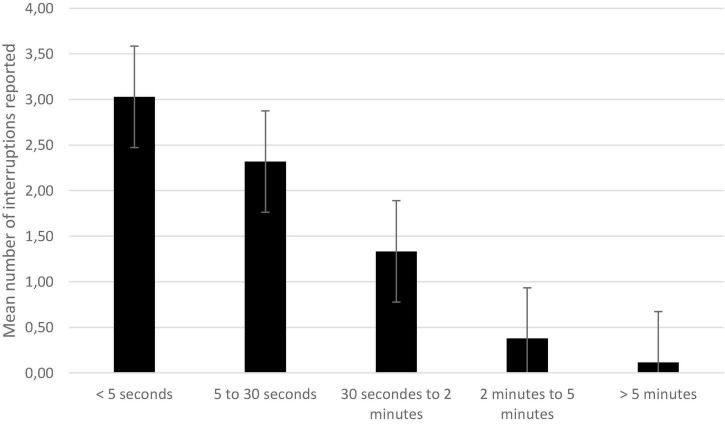
Mean number of interruptions reported as a function of duration. Error bars represent the standard errors. All comparisons are significant except the one between <5 and 5–30 s.

Post-hoc tests with Bonferroni correction revealed that even if the most frequently reported durations were less than 5 s, participants did not report significantly more interruptions lasting less than 5 s than interruptions of 5–30 s, *t*(68) = 2.08, *ns*. They mentioned more interruptions lasting between 5 and 30 s than lasting 30 s to 2 mins, *t*(68) = 3.28, *p* = 0.016. They also listed more interruptions lasting between 30 s and 2 mins than interruptions lasting 2–5 mins, *t*(68) = 5.04, *p* < 0.001. Finally, there were more interruptions lasting between 2 and 5 mins than interruptions lasting more than 5 mins, *t*(68) = 3.19, *p* = 0.021 (Figure 3).

#### Mental effort required by the interrupting task

A repeated-measures ANOVA with the number of reported interruptions as the dependent variable, and mental effort required by the interrupting task (*very low*, *low*, *moderate*, *high*, and *very high*) as within-subject factor showed a significant effect of mental effort, *F*(4, 272) = 67.80, *p* < 0.001, η^2^*_*p*_* = 0.50.

**FIGURE 4:**
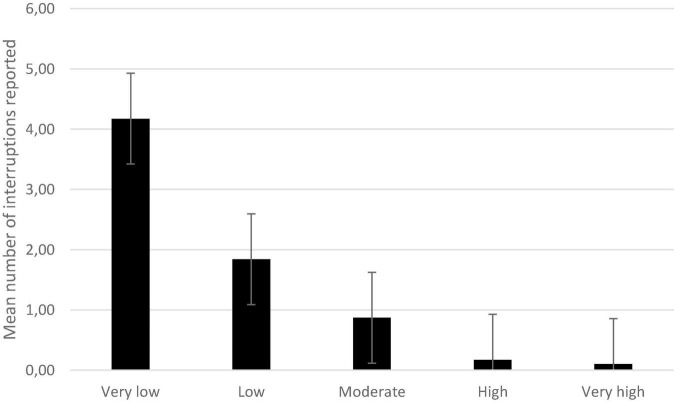
Mean number of interruptions reported as a function of reported mental effort. Error bars represent the standard errors. All comparisons are significant except the one between high and very high.

Post-hoc tests with Bonferroni correction indicated that participants reported more very low-disturbance interruptions than low-disturbance interruptions, *t*(68) = 5.82, *p* < 0.001. The differences between low- and moderate-disturbance interruptions, *t*(68) = 4.80, *p* < 0.001, and between moderate- and high-disturbance interruptions, *t*(68) = 4.27, *p* < 0.001, were also significant (Figure 4).

#### Annoyingness of interruptions

A repeated-measures ANOVA with the number of reported interruptions as the dependent variable, and the annoyingness of the reported interruption (very annoying, annoying, neutral, pleasant, and very pleasant) as the within-subject factor shows an effect of annoyingness, *F*(4, 272) = 6.32, *p* < 0.001, η^2^*_*p*_* = 0.09.

**FIGURE 5:**
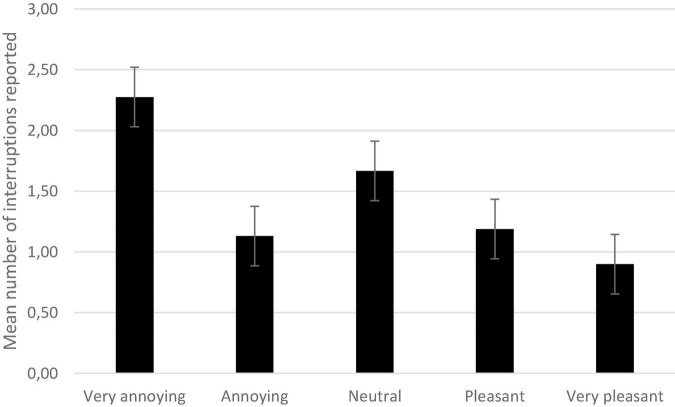
Mean number of interruptions reported as a function of their annoyingness. Error bars represent the standard errors.

Post-hoc tests with Bonferroni correction showed that participants reported more very annoying interruptions than annoying ones, *t*(68) = 3.13, *p* = 0.003. Participants also indicated more very annoying interruptions than very pleasant ones, *t*(68) = 3.80, *p* < 0.001 (Figure 5).

#### Relation between attentional disruptions and reading comprehension

We ran a multiple linear regression with total interruptions, internal interruptions, external interruptions and *mind wandering* as predictors of error rates on all questions. Contrary to our expectation, the model did not yield significant results, *F*(4, 64) < 1, *ns*. We used the same predictors for error rate on inferential questions and error rate on surface questions separately, but neither model yielded significant results, *F*(4, 64) < 1, *ns* and *F*(4, 64) < 1, *ns*, respectively.

#### Relation between attentional disruptions and reading experience

We conducted a multiple linear regression with the same predictors as listed above on the pleasure experienced during reading. The model was not significant, *F*(4, 64) < 1, *ns*. When the same predictors were used for the difficulty of reading the text, the model was once again not significant, *F*(4, 64) = 1.45, *ns*. The same predictors were used to predict the feeling of attentiveness while reading, but the model was not significant, *F*(4, 64) = 1.09, *ns*.

#### Relation between source of interruption and reading comprehension

We conducted a multiple linear regression with media-related interruptions versus media-unrelated interruptions as a predictor of the recorded error rate for all questions. The linear model was significant *F*(2, 66) = 3.88, p = 0.025 and adjusted *R*^2^ = 0.08. Among the predictors, media-unrelated interruptions reduced the error rate, *t*(66) = −2.55, *p* = 0.013 and β = −1.55.

The same predictors were used for the error rate on inferential questions; the linear model was significant, *F*(2, 66) = 3.58, *p* = 0.033 and adjusted *R*^2^ = 0.07. Among the predictors, media-unrelated interruptions reduced the error rate, *t*(66) = −2.44, *p* = 0.017, and β = −1.52.

When the same predictors were used for the error rate on surface questions, the linear model was again significant, *F*(2, 66) = 3.82, *p* = 0.037 and adjusted *R*^2^ = 0.07. Among the predictors, media-unrelated interruptions reduced the error rate, *t*(66) = −2.43, *p* = 0.018, and β = −1.58.

### Multitasking profile and reading comprehension

We divided the participants into two groups as a function of their MMI scores (Ophir et al., 2009). Participants below the median were considered as *“low multitaskers” (n* = 34) and participants who were above the median were considered as *“high multitaskers” (n* = 35). A check showed that the “*low multitaskers*” did indeed have a significantly lower MMI score (*M* = 2.41, *SD* = 0.57) than the “*high multitaskers*” (*M* = 4.33, *SD* = 0.79), *t*(67) = 11.60, *p* < 0.001, *d*′ = 2.80.

We used a repeated-measures ANOVA to compare the error rate on question type as within-subject factor (*surface* vs. *inferential*) and the level of multitasking as between-subject factor (“*low multitaskers*” vs. “*high multitasker*”). None of the effects were significant, all *p* > 0.05.

### Multitasking profile and attentional disruptions

We used a repeated-measures ANOVA to compare the number of interruptions reported as a function of the source of the interruption as within-subject factor (*media-related* vs. *media-unrelated*), and the level of multitasking as between-subject factor (“*low multitaskers*” vs. “*high multitasker*”). Despite the fact that there was no effect of the source of the interruption, *F*(1, 67) < 1, *ns*, the *“high media multitaskers”* were prone to more interruptions than the *“low media multitaskers”*, *F*(1, 67) = 4.46, *p* = 0.038, η^2^*_*p*_* = 0.06. The interaction was also significant, *F*(1, 67) = 4.23, *p* = 0.044, η^2^*_*p*_* = 0.06.

**FIGURE 6:**
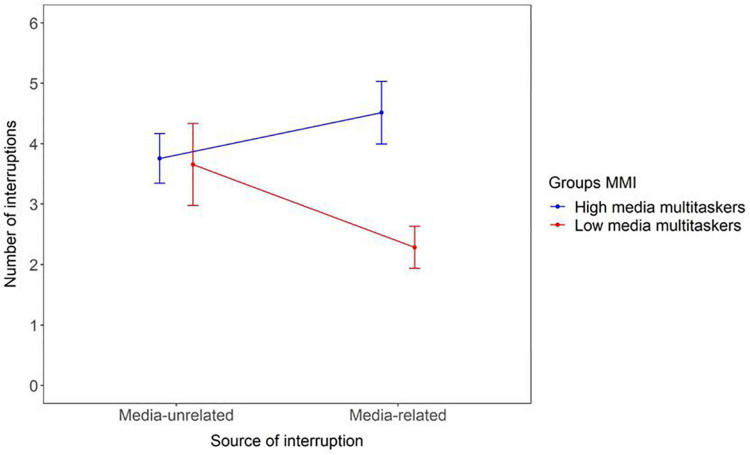
Number of interruptions reported as a function of the source of the interruption (media-unrelated or media-related) and the multitasking profile of the participants (high or low media multitaskers).

Post hoc analyses with Bonferroni correction revealed that “high media multitaskers” experienced more media-related interruptions than “low media multitaskers” *t*(67) = 3.31, *p* = 0.009, although this comparison was not significant for media-unrelated interruptions (Figure 6). This shows that the inclination to engage with multiple media as measured by the MMI was consistent with what readers reported during their reading session.

### Relation between reading comprehension and reading experience

We conducted a multiple linear regression with pleasure when reading the text, difficulty when reading the text and the feeling of attentiveness during reading as predictors of the error rate for all questions. The linear model was significant, *F*(3, 65) = 8.69, p < 0.001 and adjusted *R*^2^ = 0.25. Among the predictors, pleasure when reading the text and the feeling of attentiveness during reading were significant, respectively, *t*(65) = −2.17, *p* = 0.034, β = −5.59 and *t*(65) = −2.28, *p* = 0.026, β = −6.72.

The same predictors were used for the error rate on inferential questions and the linear model was significant, *F*(3, 65) = 6.96, p < 0.001 and adjusted *R*^2^ = 0.21. Of the predictors, pleasure when reading the text and feeling attentive while reading were significant, respectively, *t*(65) = −2.01, *p* = 0.048, β = −5.48 and *t*(65) = −2.14, *p* = 0.036, β = −6.67.

The same predictors were again used for error rate on surface questions. The linear model proved to be significant, *F*(3, 65) = 8.92, p < 0.001 and adjusted *R*^2^ = 0.26. Among the predictors, pleasure when reading the text and feeling of attentiveness during reading were significant, respectively, *t*(65) = −2.10, *p* = 0.040, β = −5.73 and *t*(65) = −2.16, *p* = 0.034, β = −6.77.

### Relation between attentional disruption and self-evaluation

We ran a multiple linear regression with total interruptions, internal interruptions, external interruptions and *mind wandering* as predictors of the difference between actual score on comprehension question and estimated score to comprehension question. The model was not significant, *F*(4, 64) < 1, *ns*.

## Discussion

This study was carried out to investigate attentional disruptions that may occur in an ecological context during reading on a computer screen and their impact on reading comprehension and the reading experience. We hypothesized that there would be a negative correlation between the number of interruptions and the scores on inferential questions, but not on factual information. Contrary to our expectations, the number of interruptions was not related to performance on inferential question. In addition, we also investigated whether our participants’ chronic inclination toward media multitasking was related to the characteristics of these interruptions. We expected the *high media multitaskers* to experience more media-related interruptions during the experimental reading session. The interaction between the source of the interruptions and the level of multitasking validates this hypothesis. An analysis revealed no relation between attentional disruption and the pleasure experienced when reading the text. However, our data show a positive correlation between pleasure when reading the text and reading comprehension.

Our study reveals very frequent attentional disruptions during a computer reading session in an ecological context. Considering all the types of attentional disruptions measured in our study (i.e., interruptions and *mind wandering*), participants reported a diversion of attention away from the text every 4 mins on average. Interestingly, these attentional disruptions occurred more frequently in the first half of the text. This could be explained by the increasing involvement of the reader in the story. As the reader progressed through the story, the richer the situation model became (Smallwood et al., 2008) and the more likely it is that the reader’s interest in the text increased. Previous research has shown that interest in the task reduces the willingness to interrupt it (Deng, 2020). This could explain the reduction of both *mind wandering* and interruptions. To test this hypothesis, it might be interesting to assess readers’ interest as they progress through the text and observe whether it increases as the frequency of attentional disruptions decreases.

Interestingly, there was no difference between the number of interruptions initiated by the participants and interruptions triggered by an external source. It is possible that the participants needed some breaks in their reading, and when the breaks were not provoked by external sources, they introduced the breaks themselves. This hypothesis should be tested by comparing two groups of participants free to interrupt themselves during a reading task, with one of the groups also being subject to external interruptions. Participants in the group exposed to interruptions should interrupt themselves less frequently. In our study, neither internal interruptions nor external interruptions were related to reading comprehension.

Attentional disruptions did not impact comprehension in response to either surface or inferential questions. If we think of interruptions as situations in which the participant must interrupt the reading task to do something else, these results are not particularly surprising. Most studies that have investigated interruptions have not shown an effect on reading comprehension (Glanzer et al., 1981, 1984; Glanzer and Nolan, 1986). This is consistent with the predictions of long-term working memory theory. According to this theory, once information is read, it is integrated into a situation model in long-term memory. This prevents the degradation of information in memory (Ericsson and Kintsch, 1995; Delaney and Ericsson, 2016). Another explanation might be that participants re-read the text preceding the interruptions so that they can reintegrate the missing information into their situation model, as has previously been shown in laboratory studies (Cane et al., 2012; Chevet et al., 2021). Such a strategy is likely to increase total reading time (Fox et al., 2008; Bowman et al., 2010), but is effective in preserving comprehension. The lack of effect of interruptions on comprehension could also be related to the characteristics of the interruptions themselves. Indeed, most of the reported interruptions were of very short duration and required very little mental effort to process. It is therefore likely that they did not disturb the readers to the point of reducing their comprehension of the text. To our knowledge, Oulasvirta and Saariluoma (2006) are the only authors who have directly manipulated the difficulty of the interrupting task, and they found no decrease in comprehension due to a highly demanding interrupting task. However, in their study, the participants did not rate the difficulty of the interrupting task. The interrupting task was thought to be either more or less difficult than the experimental manipulation performed in a previous study (Byrne and Anderson, 2001). Further studies should assess both the perceived difficulty of the interrupting task performance on this task.

Interestingly, comprehension performance increased with the number of media-unrelated but not media-related interruptions. This positive influence of media-unrelated interruptions on comprehension could be explained by rereading behaviors when the participants resumed reading. This type of strategy leads to the repeated encoding of information. Nevertheless, this explanation could also apply to media-related interruptions. In a laboratory experiment, it might be interesting to manipulate the source of the interruption to see if media-related interruptions are less likely to result in rereading behaviors than media-unrelated interruptions.

Regarding self-reported mind wandering, previous research has shown that it is not necessarily related to reading comprehension (Schooler et al., 2004), as was the case in our study. As may happen after an interruption, participants who realize that their attention is no longer focused on the text may implement a backtracking strategy to regain the reading thread by rereading the text (Varao-Sousa and Kingstone, 2019; Cane et al., 2012; Chevet et al., 2021). Although it might appear difficult to do in practice, it would be interesting to collect information on the proportion of unconscious *mind wandering* in an ecological situation. Indeed, this type of disruption does not allow for a rereading strategy and has been linked to decreased comprehension in the laboratory (Schooler et al., 2004; Smallwood et al., 2008). The use of an external probe to gather information could be considered, but this necessarily leads to an impairment of the ecological nature of the task. A different solution could be to use an *eye-tracking* device to detect *mind wandering* (Reichle et al., 2010; Faber et al., 2018; Brishtel et al., 2020). Indeed, eye movements are different in mindless reading (fewer and longer fixations, greater variability in pupil diameter). Such devices are non-invasive and permit a good estimation of *mind wandering* without interrupting the reader. However, although it is possible to use them for laboratory studies, current technology does not make this possible in an ecological situation.

Our data suggest a relationship between the feeling of attentiveness while reading and comprehension on both types of questions. Specifically, the more attentive participants felt during reading, the better they performed on the comprehension test. This is somewhat surprising because the attentional disruptions actually reported by participants during the reading session were not related to comprehension. A possible explanation could be that when assessing the feeling of attentiveness after reading, participants realized that they were not attentive at certain points during their reading but without having identified and reported this at the time. Such situations could correspond to unconscious *mind wandering* episodes encountered during the reading session, further emphasizing the importance of assessing them in future research.

The pleasure experienced when reading the text was also positively correlated with comprehension. This result is consistent with the fact than the motivation to read is related to comprehension (Guthrie and Cox, 2001). However, pleasure when reading the text was not affected by attentional disruptions. This could be related to the mixed results regarding the pleasure experienced as a result of the interruptions. Indeed, although most of the interruptions were considered annoying and may have decreased satisfaction during the reading session, some were actually enjoyable for the participants.

Finally, and as expected, the *high media multitaskers* were more often interrupted by media than the *low media multitaskers* during their reading sessions, thus leading to a greater number of total interruptions for the *high media multitaskers*. This result argues in favor of the methodology we used to identify *high* and *low media multitaskers*. Our data suggest that the inclination to use multiple media simultaneously led to more interruptions during a reading session. It might be interesting to extend these results to activities other than reading in order to assess the extent to which the tendency to use multiple media simultaneously results in more interruptions in daily activities.

For future research, it could be interesting to collect information not only related to interruptions occurring in an ecological environment of reading, but also to collect data about the home environment itself (set up of the computer, presence of other people or pet around during the reading); and also, more participants’ characteristics (reading level, attitude toward reading in general) in order to investigate a potential relation with the number of interruptions. Additionally, instead of threshold for duration of the interruptions, it would be a very interesting approach to stopwatch it in further study. This could allow a better interpretation of the impact of these interruptions on the reading time. Also, the completion of the grid for interruption adds additional time to the interruption; we encourage future research to propose alternative solution.

## Conclusion

In conclusion, this study provides useful information on the characteristics of attentional disruptions likely to occur during a computer reading session in an ecological context. This study shows clearly that interruptions and attentional disruptions are frequent during reading in digital-based and ecologic situations. It also confirms that a chronic inclination toward media multitasking in daily activities leads to more media-related interruptions during a reading session. However, when they are media-unrelated, interruptions are associated with better comprehension. Therefore, in the context of learning it might be necessary to avoid the superposition of media-related activities, especially when they are not required for the activity, while allowing some breaks likely to bring a reinvestment of attentional resource.

## Data availability statement

The raw data supporting the conclusions of this article will be made available by the authors, without undue reservation.

## Ethics statement

Ethical review and approval was not required for the study on human participants in accordance with the local legislation and institutional requirements. Written informed consent for participation was not required for this study in accordance with the national legislation and the institutional requirements.

## Author contributions

GC and VD-Z contributed to the conception and design of the study and wrote the first draft of the manuscript. GC organized the database. GC, VD-Z, and TB performed the statistical analysis. All authors contributed to manuscript revision, read, and approved the submitted version.
