# Corrigendum: What breaks the flow of reading? A study on characteristics of attentional disruption during digital reading

**DOI:** 10.3389/fpsyg.2022.1109893

**Published:** 2022-12-13

**Authors:** Guillaume Chevet, Thierry Baccino, Annie Vinter, Véronique Drai-Zerbib

**Affiliations:** ^1^Laboratory LEAD-CNRS, UMR5022, Université Bourgogne, Dijon, France; ^2^LUTIN, University Paris 8, Paris, France

**Keywords:** reading, attentional disruption, interruption, mind wandering, multitasking

In the original article, there was an error in the correspondence details listed. Instead of “Guillaume Chevet and Thierry Baccino,” the two corresponding authors should be “Guillaume Chevet and Véronique Drai-Zerbib.” The corrected correspondence details are shown below.

^*^Correspondence:

Guillaume Chevet

guillaume.chevet@u-bourgogne.fr

Véronique Drai-Zerbib

veronique.drai-zerbib@u-bourgogne.fr

The authors apologize for this error and state that this does not change the scientific conclusions of the article in any way. The original article has been updated.

